# Impact of residual tricuspid regurgitation after transcatheter tricuspid therapies on right and left ventricular mechanics: insights from a case series

**DOI:** 10.1093/ehjcr/ytag626

**Published:** 2026-09-24

**Authors:** Giulio M Mondellini, Antoon J M van den Enden, Mark M P van den Dorpel, Christiaan L Meuwese, Nicolas M Van Mieghem

**Affiliations:** Department of Cardiology, Erasmus University Medical Center (Erasmus MC), Dr. Molewaterplein 40, 3015 GD Rotterdam, The Netherlands; Department of Cardiology, Erasmus University Medical Center (Erasmus MC), Dr. Molewaterplein 40, 3015 GD Rotterdam, The Netherlands; Department of Cardiology, Erasmus University Medical Center (Erasmus MC), Dr. Molewaterplein 40, 3015 GD Rotterdam, The Netherlands; Department of Cardiology, Erasmus University Medical Center (Erasmus MC), Dr. Molewaterplein 40, 3015 GD Rotterdam, The Netherlands; Department of Intensive Care Adults, Erasmus University Medical Center (Erasmus MC), Dr. Molewaterplein 40, 3015 GD Rotterdam, The Netherlands; Department of Cardiology, Erasmus University Medical Center (Erasmus MC), Dr. Molewaterplein 40, 3015 GD Rotterdam, The Netherlands

**Keywords:** Pressure-volume loop, Haemodynamics, Valve replacement, Edge-to-edge repair, Transcatheter interventions, RV function, Case series

## Abstract

**Background:**

Severe tricuspid regurgitation (TR) induces right ventricular (RV) volume overload and disrupts biventricular mechanics. Tricuspid transcatheter edge-to-edge repair (T-TEER) and transcatheter tricuspid valve replacement (TTVR) are effective strategies to reduce or eliminate TR, yet their immediate physiological effects and the impact of residual TR remain incompletely characterized. We used invasive pressure-volume (PV) analysis to assess acute biventricular responses to transcatheter tricuspid valve interventions.

**Case summary:**

Three patients with severe TR underwent TTVR (*n* = 1) or T-TEER (*n* = 2) with invasive RV and left ventricular (LV) PV assessment immediately before and after intervention. In two patients achieving ≤mild residual TR, RV end-diastolic volume decreased (241→205 mL; 81→77 mL), while RV end-systolic pressure increased (32→37 mmHg; 26→32 mmHg). Effective arterial elastance (Ea) increased markedly (0.28→0.50; 0.84→1.33 mmHg/mL), paralleled by increases in end-systolic elastance (Ees) (0.26→0.38; 0.49→0.80 mmHg/mL), consistent with an adaptive contractile response. These changes were accompanied by increased LV end-diastolic volumes (163→176 mL; 68→84 mL) and higher LV/RV volume ratios. In contrast, in the patient with residual moderate-to-severe TR, only modest changes in RV mechanical indices were observed, without an increase in LV preload (102→99 mL).

**Discussion:**

Effective TR reduction appears to be a key determinant of acute biventricular adaptation following transcatheter tricuspid valve interventions. Optimal reduction (<moderate TR) is associated with RV volume unloading, increased afterload, and a coupled rise in contractile indices, resulting in improved LV filling. In contrast, suboptimal TR reduction yields limited biventricular benefit.

Learning points

- Transcatheter repair or replacement for severe TR reduces RV volume loading and increases afterload (Ea) with parallel changes in contractile indices (Ees), while increasing LV preload only with optimal TR reduction.
- These findings may provide a pathophysiological explanation for the symptomatic benefits observed with effective TR reduction in clinical trials.

## Introduction

Severe tricuspid regurgitation (TR) generates volume overloading of the right ventricle that affects right and left ventricular (RV and LV) cardiac mechanics and is associated with adverse clinical outcomes.^1^ Tricuspid transcatheter edge-to-edge repair (T-TEER) and transcatheter tricuspid valve replacement (TTVR) have emerged as attractive strategies to reduce or eliminate TR.

More than mild residual TR is relatively common after T-TEER but rare after TTVR, which is less affected by anatomical variability and complexity of the tricuspid valve (e.g. coaptation gaps, excessive leaflet tethering, unfavourable jet locations). In the randomized controlled TRILUMINATE pivotal and Tri.Fr trials that compared T-TEER with standard of care, the occurrence of moderate or greater residual TR after T-TEER was 50% and 60%, respectively.^2,3^ In the TRISCEND-II trial that compared TTVR with standard of care, 95.3% of patients had ≤mild residual TR after TTVR.^4^

The effects of T-TEER and TTVR on cardiac mechanics remain elusive. The invasive assessment of RV and LV pressure-volume relationships provides real-time monitoring of cardiac mechanics.^5^ In this case series of patients with severe TR, we aimed to describe the immediate effects of T-TEER and TTVR on RV and LV pressure-volume relationships and to assess the impact of residual TR.

### Methods

We included three patients with severe TR who underwent T-TEER or TTVR and provided written informed consent for the procedure and participation in the PLUTO-II study (*Real time Pressure volume Loop monitoring as a guide for enhanced Understanding of changes in elemental cardiovascular physiology during Therapeutic strategies for hemodynamic Optimization, Cohort II: Structural Heart Interventions, NCT06204783*).^6^ All patients were treated under general anaesthesia and transoesophageal echocardiography guidance. A 7F conductance catheter (CD Leycom, Hengelo, The Netherlands) was used to obtain invasive pressure-volume relationships in the RV and LV before and after the tricuspid valve intervention (TVI).^6,7^

Prior to sampling of the PV loops, RV and LV end-systolic and end-diastolic volumes were measured by 3D transoesophageal echocardiography (Philips EPIQ Ultrasound system, Philips Healthcare, Andover, Massachusetts, USA or Siemens Acuson SC2000 Prime, Siemens Healthineers AG, Forchheim, Germany) for volume calibration, before and immediately after device deployment and haemodynamic stabilization. Single-beat estimations were used to derive the end-systolic elastance (Ees), measure of the load-independent contractility.^8–10^ Ventricular afterload was quantified by the effective arterial elastance (Ea), which is the ratio of ESP/SV.

Tricuspid regurgitation severity was assessed intraoperatively by experienced echocardiography specialists according to European Society of Cardiology guidelines,^11^ with a multiparametric approach.

An overview of baseline clinical characteristics, haemodynamic data, and echocardiographic parameters for all patients is provided in *Table 1*.

**Table 1:** Demographics and baseline characteristics

	Patient 1	Patient 2	Patient 3
Demographics			
Age, years	77	78	79
Sex	Female	Female	Male
BMI, kg/m^2^	22.86	25.28	28.41
NYHA functional class	IV	III	III
TRI-SCORE	6	3	4
Past medical history			
Arterial hypertension,	●	●	○
Chronic kidney disease^a^	●	●	●
Diabetes mellitus	○	○	○
Atrial fibrillation	●	●	●
Stroke or TIA	○	○	○
Coronary artery disease	○	○	●
Pacemaker lead	●	○	●
COPD	○	○	○
Malignancy	○	○	○
Laboratory			
Haemoglobin, mmol/L	5.9	8.4	8.2
e-GFR (mL/min/1.73 m^2^)^b^	65	50	58
NT-proBNP, pmol/L	539	25	158
White blood cell count, ×10^9^/L	8.4	7.7	5.0
Total bilirubin, µmol/L	10	7	12
Baseline echocardiography			
Tricuspid regurgitation severity	●	●	●
Tricuspid regurgitation aetiology	V-STR	A-STR	A-STR
Vena contracta width, mm	15	11	9
CWD contour	Dense, triangular	Dense, parabolic	Dense, parabolic
Hepatic vein systolic flow reversal	●	●	●
LVEF, (%)	45	45	50
TAPSE, mm	13	22	15
S′ wave TDI, cm/s	10	12	10
RV end-diastolic diameter, mm	52	38	43
Mitral regurgitation	Mild	Mild	Mild
Baseline right heart catheterization			
Systolic PA pressure, mmHg	34	27	41
Diastolic PA pressure, mmHg	12	14	19
Mean PA pressure, mmHg	20	19	25
Pulmonary capillary wedge pressure, mmHg	14	5	14
Cardiac output, L/min	4.7	2.0	3.6
Heart rate, beats/min	60	58	78

○ indicates an absent condition; ● indicates a present condition.

BMI, body mass index; COPD, chronic obstructive pulmonary disease; LVEF, left ventricular ejection fraction; e-GFR, estimated glomerular filtration rate; PA, pulmonary artery; PG, pressure gradient; RVEDD, RV end-diastolic diameter; NYHA, New York Heart Association; TAPSE, tricuspid annular plane systolic excursion; TDI, tissue Doppler imaging; A-STR, atrial secondary tricuspid regurgitation; V-STR, ventricular secondary tricuspid regurgitation.

^a^Chronic kidney disease was graded according to the 2015 Kidney Disease Improving Global Outcomes definition.

^b^e-GFR was calculated according to Chronic Kidney Disease Epidemiology Collaboration (CDK-EPI) principles.

## Summary figure

**Figure:**
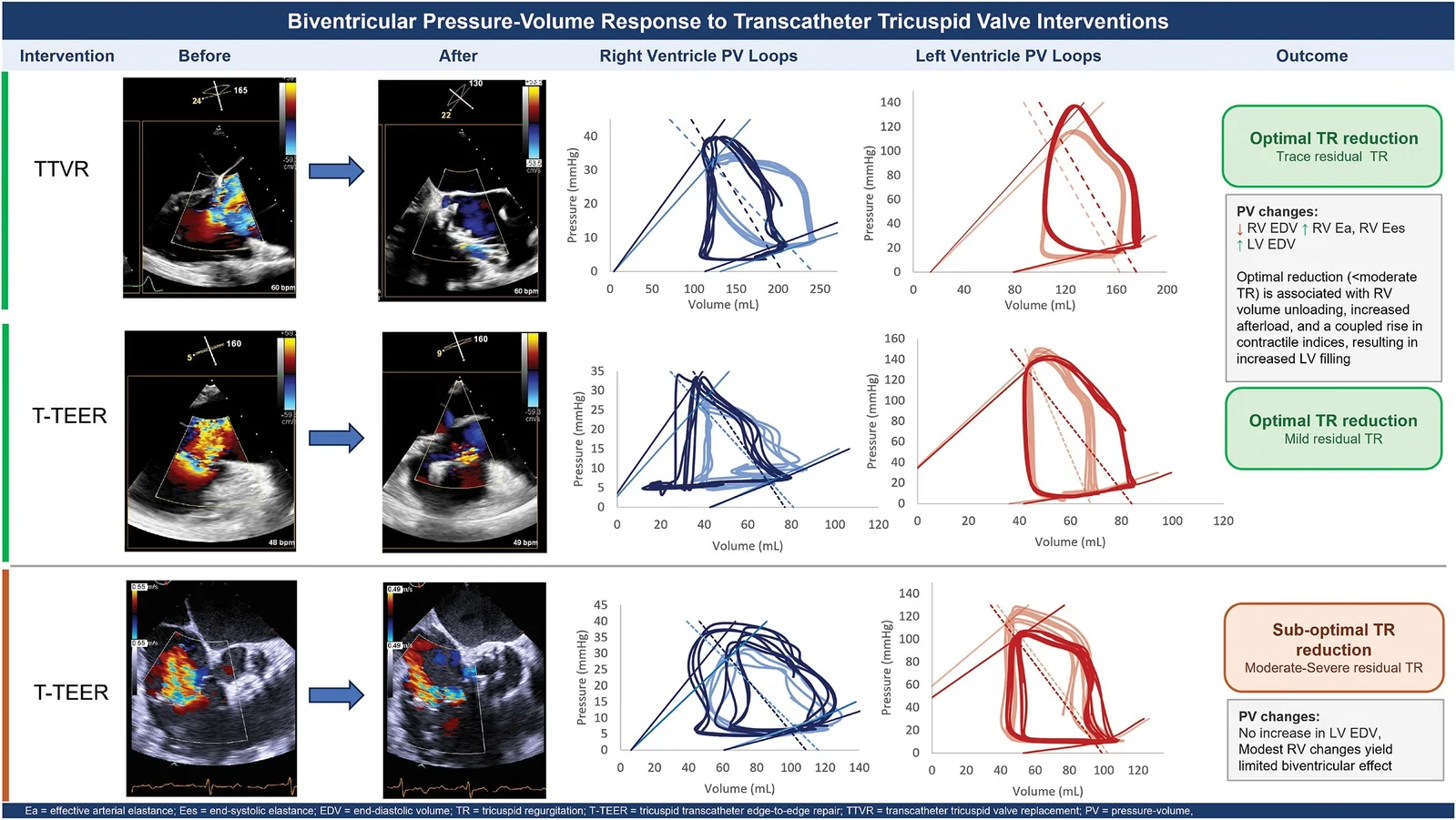
Biventricular pressure-volume adaptation following transcatheter tricuspid valve intervention according to residual tricuspid regurgitation severity.

## Patient 1

Patient 1 was a 77-year old female with a history of atrial fibrillation (AF) and permanent pacemaker implantation who presented in New York Heart Association class (NYHA) IV. Echocardiography revealed an LV ejection fraction (EF) of 45%, a dilated RV (end-diastolic diameter, EDD 52 mm) with mild RV dysfunction (reduced longitudinal contraction (TAPSE 13 mm, S′ wave TDI 10 cm/s), diastolic leftward shift of the interventricular septum, and massive ventricular secondary TR with annular dilatation. She underwent a successful *trans*-jugular TTVR with the LuX Valve (Jenscare Scientific, Ningbo, China) with a trace residual paravalvular TR and a mean gradient of 2 mmHg, immediately after the procedure under general anaesthesia and 3 mmHg at discharge. The post-procedural course was notable for minor jugular access site bleeding, without further complications, and the patient was discharged on post-operative day (POD) 2.

**Figure 1:**
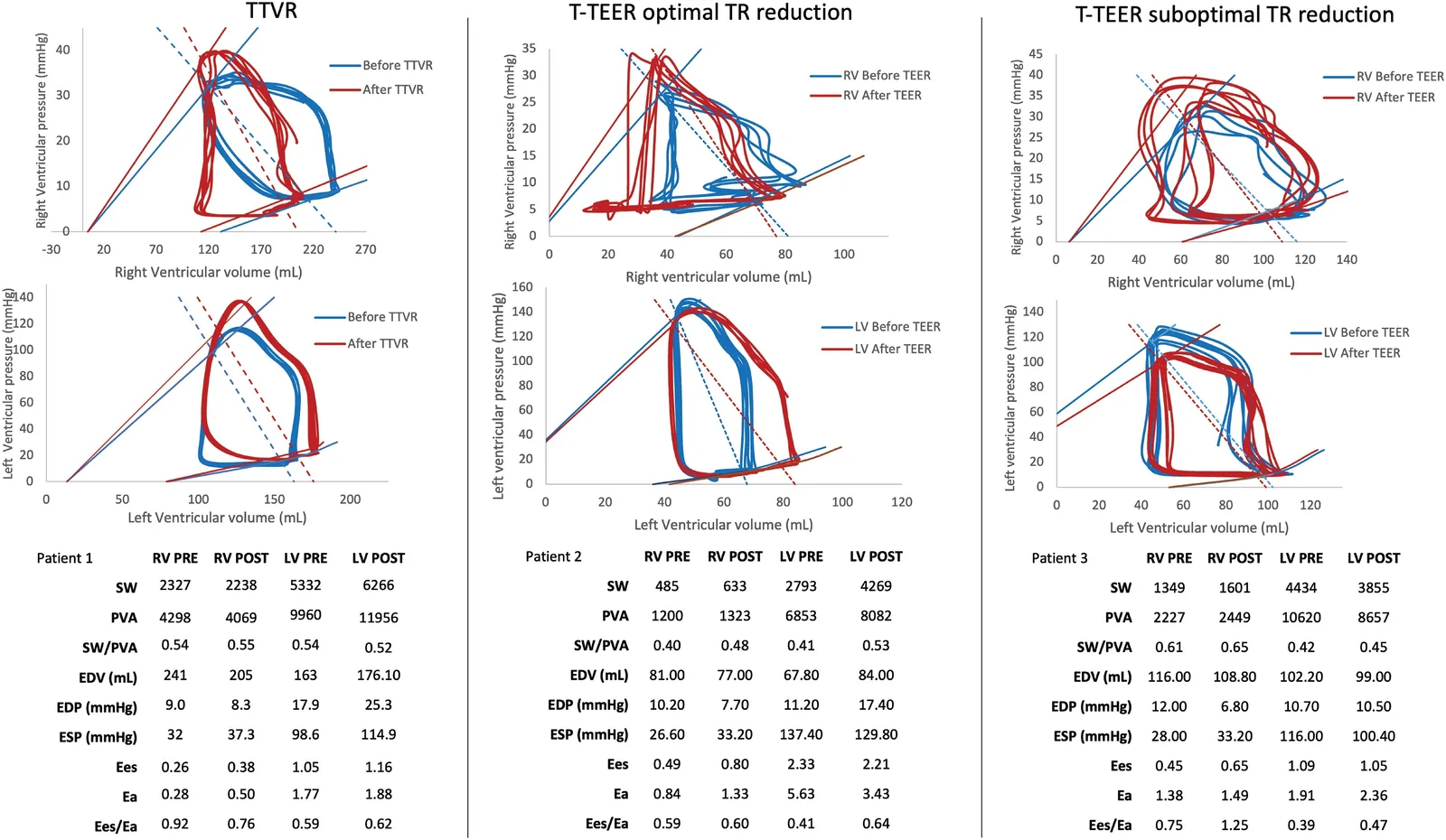
RV and LV PV reconstructions before and after TTVR (left), before and after TEER with optimal TR reduction (centre), and suboptimal TR reduction (right). These PV loops appraise the immediate changes in cardiac mechanics induced by tricuspid valve interventions for severe TR, based on conductance catheter measurements. Ventricular contractile properties are described by the end-systolic PV relationship (ESPVR) and the diastolic function is described by the end-diastolic PV trendline. Systolic function can be summarized by the slope of ESPVR (known as end-systolic elastance, Ees). Afterload can be characterized by the effective arterial elastance (Ea, dashed lines).

*Figure 1* illustrates the invasively measured pressure-volume relationships of the RV and LV before and after the tricuspid valve interventions. The RV end-diastolic volume (EDV) decreased from 241 to 205 mL and end-systolic pressure (ESP) increased from 32 to 37 mmHg. Effective arterial elastance (Ea) almost doubled from 0.28 to 0.50 mmHg/mL, Ees concomitantly increased from 0.26 to 0.38 mmHg/mL, and Ees/Ea ratio changed from 0.92 to 0.76, whereas the load-dependent dP/dt_max_ from 269.8 to 179.3 mmHg/s, likely influenced by the change in loading conditions after the intervention. Stroke work (SW) to pressure-volume area (PVA) remained stable. Left ventricular EDV increased from 163 to 176 mL. The ratio of LV EDV to RV EDV increased from 163/241 (0.68) to 176/205 (0.86).

## Patient 2

**Figure 2:**
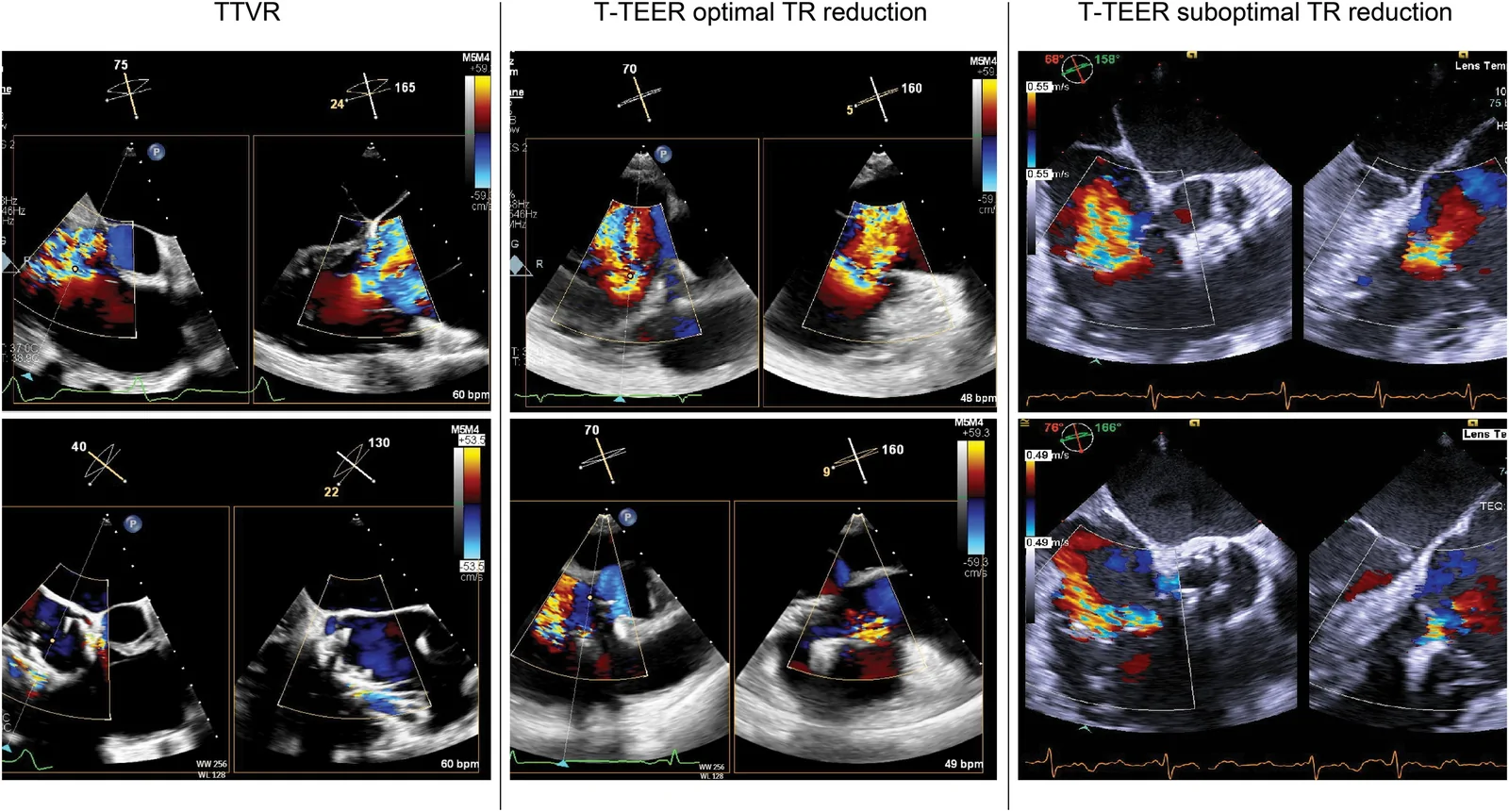
Colour-Doppler imaging in transoesophageal echocardiography before (top panels) and after (bottom panels) tricuspid valve interventions for severe TR. Lower panels illustrate the changes after the interventions: TTVR (left), tricuspid TEER with optimal TR reduction (centre), and TEER with suboptimal TR reduction (right).

Patient 2 was a 78-year-old female with a history of hypertension and AF presented in NYHA class III. Echocardiography revealed an LVEF 45%, preserved RV systolic function (TAPSE 22 mm, S′ wave TDI 12 cm/s), RVEDD 38 mm, right atrial enlargement, annulus dilatation and severe atrial-secondary TR. T-TEER with 2 TriClip devices (Abbott Vascular (Abbott, Chicago, IL, USA) resulted in mild residual TR (*Figure 2*), with a mean tricuspid gradient of 1 mmHg, confirmed at discharge. Right ventricular EDV decreased from 81 to 77 mL, ESP increased from 26 to 32 mmHg, Ea increased from 0.84 to 1.33 mmHg/mL, and Ees concomitantly increased from 0.49 to 0.80 mmHg/mL. The Ees/Ea ratio remained stable (0.59 and 0.60), dP/dt increased from 123 to 148 mmHg/s, and SW/PVA increased from 0.40 to 0.48. LV EDV increased from 68 to 84 mL. The LV/RV EDV ratio increased from 68/81 (0.84) to 84/77 (1.09). The post-procedural course was uneventful, and the patient was discharged on POD 1.

## Patient 3

Patient 3 was a 79-year-old-male, with AF, ischaemic cardiomyopathy, and permanent pacemaker who presented in NYHA class III. Echocardiography revealed an LVEF 50%, mild RV impairment (TAPSE 15 mm, S′ wave TDI 10 cm/s), RV EDD 43 mm, and right atrial dilation, with severe atrial secondary TR. After T-TEER with 2 TriClips, there was moderate-severe residual TR (*Figure 2*) and a mean tricuspid gradient of 1 mmHg (2 mmHg at discharge). The RV pressure-volume relationship exhibited changes of RV mechanics in terms of Ea and Ees, without meaningful effects on LV preload (EDV from 102 to 99 mL). The patient was discharged in POD 2, after a regular post-operative course.

In-hospital and 30-day clinical outcomes following transcatheter tricuspid valve interventions are summarized in *Table 2*.

**Table 2:** In hospital outcomes and 30-day follow-up after transcatheter tricuspid valve interventions

	Patient 1	Patient 2	Patient 3
In-hospital outcomes			
Residual TR severity at discharge	Trace	Mild	Moderate-severe
Mean atrioventricular PG, mmHg at discharge	3	1	2
Acute kidney injury^a^	○	○	○
Bleeding complications^b^	○	○	○
Vascular complications^c^	Minor	○	○
30-day follow-up			
All-cause mortality	○	○	○
NYHA class	II	II	III
Stroke or TIA	○	○	○
Myocardial infarction	○	○	○
New PPM	○	○	○
HF rehospitalization	○	○	○

○ indicates absence of the condition; ● indicates presence of the condition.

HF, heart failure; LVEF, left ventricular ejection fraction; MG, mean gradient; NYHA, New York Heart Association; PG, peak gradient; PPM, permanent pacemaker; TIA, transient ischaemic attack.

^a^Acute kidney disease was graded according to the 2012 Kidney Disease Improving Global Outcomes definition.

^b^Defined as Bleeding Academic Research Consortium (BARC) type of at least 2.

^c^Defined according to TVARC Tricuspid Valve Academic Research Consortium guidelines.

## Discussion

In this case series, we describe the effects of different tricuspid interventions to treat severe TR on RV and LV pressure-volume relationships. In the TTVR and T-TEER cases that resulted in <moderate residual TR, immediate volume unloading of the RV was associated with relevant increases in Ea and Ees and LV EDV. In contrast, in the patient with more than moderate residual TR after tricuspid TEER, only modest changes in RV mechanical indices were observed, without a meaningful increase in LV preload, underscoring the limited biventricular impact of suboptimal TR reduction.

In patients with severe TR, successful reduction to <moderate residual TR had consistent effects on RV and LV cardiac mechanics, regardless of transcatheter repair or replacement strategy. In TTVR and TEER with optimal TR reduction, we observed a >50% increase in RV (Ea) and almost similar increases (Ees). Incremental arterial elastance (Ea), a lumped parameter of downstream pressure and intrinsic properties of the ventricle and pulmonary vasculature, reflects higher RV afterload,^5^ whereas the higher load-independent RV Ees reflects an increased myocardial contractile state.^5^ The observed increased Ees in parallel with Ea is consistent with an adaptive ventricular contractile response after successful TR reduction and it might be explained by the Anrep effect, which is a ‘homeometric’ autoregulation mechanism^12^ that consists of the recruitment of dormant myosin heads to catalyse actin-myosin crossbridging and preserve stroke volume and cardiac output in response to acutely increased afterload.^13^ The observation that the effects on RV Ees after successful T-TEER and TTVR were similar in response to increased afterload is intriguing. It might suggest that expanding an oversized frame within the tricuspid annulus does not necessarily impair RV contractility. However, confirmation in larger cohorts is warranted.

Significant TR reduction to <moderate TR increases the forward flow from the RV towards the LV, with resultant increase in LV EDV. Incremental LV preload was well tolerated and associated with stable Ees/Ea ratio (i.e. LV-aortic coupling). Sufficient TR reduction is required to achieve favourable effects of transcatheter therapies on RV and LV mechanics. These observations may provide a pathophysiological explanation for the effects of T-TEER on functional status in clinical trials. In the TRILUMINATE pivotal trial,^2^ T-TEER only had meaningful improvements in quality of life when the residual TR was ≤moderate. Impact of T-TEER on QoL metrics was also more robust with lower grades of residual TR at 1-year follow-up in Tri.Fr trial.^3^ In addition, residual moderate TR (2+) was found to be an independent predictor for recurrent severe or worse TR, at 1 year in a multicentre retrospective registry.^14^

To our knowledge, this study represents the first report of invasive biventricular PV responses following different transcatheter TVI. A few limitations should be acknowledged. Single-beat algorithms have limited validity in the context of structural interventions, and absolute values of Ees and Ees/Ea should therefore be interpreted with caution. Our findings reflect acute physiological responses in a selected patient cohort rather than definitive measures of ventricular contractile performance. In addition, LV and RV assessments were not performed simultaneously. In the intraprocedural setting, detailed quantitative evaluation was not performed. Nevertheless, TR was assessed by experienced imaging cardiologists with a multiparametric approach.

All patients were treated under general anaesthesia, and the use of sedatives and vasoactive substances may represent uncontrollable confounders that should be considered when interpreting our observations. Although pre- and post-TVI acquisitions were performed under comparable conditions in terms of mechanical ventilation, anaesthetics, and vasoactive drug dosages, the cumulative effect of these agents cannot be excluded. Because of these limitations, our study should be considered hypothesis-generating.

## Conclusions

Transcatheter valve repair or replacement for severe TR reduces RV volume loading and increases RV afterload and contractility and LV preload only with sufficient TR reduction. These findings likely represent acute haemodynamic adaptations to restoration of tricuspid valve competence and should be interpreted as preliminary mechanistic observations that warrant confirmation in larger cohorts.

## Lead author biography


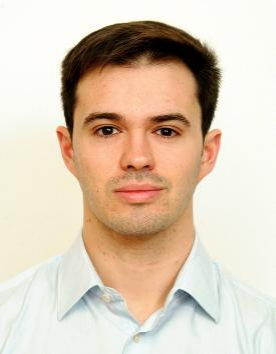


Dr Giulio Mondellini is a cardiologist and PhD candidate at Erasmus MC, Rotterdam, the Netherlands. He completed his cardiology residency at Università degli Studi di Milano. His clinical and research interests focus on haemodynamics of valvular heart disease, heart failure, and mechanical circulatory support. His research aims to enhance understanding of mechanisms in structural heart disease and improve therapeutic strategies for patients with complex cardiovascular conditions.

## Data Availability

Raw data are available upon reasonable request to the corresponding author.
